# *In silico* regenerative medicine: how computational tools allow regulatory and financial challenges to be addressed in a volatile market

**DOI:** 10.1098/rsfs.2015.0105

**Published:** 2016-04-06

**Authors:** L. Geris, Y. Guyot, J. Schrooten, I. Papantoniou

**Affiliations:** 1 Prometheus, Division of Skeletal Tissue Engineering, KU Leuven, Onderwijs en Navorsing 1 (+8), Herestraat 49-PB813, Leuven 3000, Belgium; 2 Skeletal Biology and Engineering Research Center, KU Leuven, Onderwijs en Navorsing 1 (+8), Herestraat 49-PB813, Leuven 3000, Belgium; 3 Biomechanics Research Unit, Université de Liège, Chemin des Chevreuils 1 - BAT 52/3, Liège 4000, Belgium; 4 Department of Mechanical Engineering, Biomechanics Section, KU Leuven, Celestijnenlaan 300C-PB 2419, Leuven 3001, Belgium; 5 Antleron BVBA, Leuven, Belgium

**Keywords:** regenerative medicine, tissue engineering, bioprocess, bioreactor, *in silico* model

## Abstract

The cell therapy market is a highly volatile one, due to the use of disruptive technologies, the current economic situation and the small size of the market. In such a market, companies as well as academic research institutes are in need of tools to advance their understanding and, at the same time, reduce their R&D costs, increase product quality and productivity, and reduce the time to market. An additional difficulty is the regulatory path that needs to be followed, which is challenging in the case of cell-based therapeutic products and should rely on the implementation of quality by design (QbD) principles. *In silico* modelling is a tool that allows the above-mentioned challenges to be addressed in the field of regenerative medicine. This review discusses such *in silico* models and focuses more specifically on the bioprocess. Three (clusters of) examples related to this subject are discussed. The first example comes from the pharmaceutical engineering field where QbD principles and their implementation through the use of *in silico* models are both a regulatory and economic necessity. The second example is related to the production of red blood cells. The described *in silico* model is mainly used to investigate the manufacturing process of the cell-therapeutic product, and pays special attention to the economic viability of the process. Finally, we describe the set-up of a model capturing essential events in the development of a tissue-engineered combination product in the context of bone tissue engineering. For each of the examples, a short introduction to some economic aspects is given, followed by a description of the *in silico* tool or tools that have been developed to allow the implementation of QbD principles and optimal design.

## 1. Introduction

Regenerative medicine refers to the branch of medicine that attempts to replace or regenerate human cells, tissues or organs in order to restore or establish normal function [1]. It uses a combination of several technological approaches that moves it beyond traditional transplantation and replacement therapies. These approaches may include, but are not limited to, the use of soluble molecules, gene therapy, stem cell transplantation, tissue engineering (TE) and the reprogramming of cell and tissue types [2].

Regenerative medicine has the potential to emerge as a major growth driver of the global economy, a potential that is demonstrated by the worldwide TE and cell therapy market [3]. The road to maturation of this cell-based regenerative medicine industry is becoming clear through the high number of clinical development activities and the growing interest of big pharma and medical device companies [3]. Venture capital investors also see near-term revenue opportunities for companies making tools for the industry or using stem cells for drug discovery and development. Despite these drivers, the regenerative medicine market still remains in its commercial infancy because advanced therapeutic medicinal products (ATMPs) in general, and cell-based combination products (combinations of carriers and cells) in particular, represent new technology and business models that are both different from traditional drug or device development. Furthermore, start-up biotech and cell therapy companies lack the financial means and the clinical, regulatory and manufacturing capabilities necessary to establish a product portfolio and technology pipeline. The high costs and lack of awareness remain the main restraints for the use of cell-based combination products. This is illustrated by the fact that up to date only five cell-based products have been approved by the European Medicines Agency (http://www.ema.europa.eu/ema/index.jsp?curl=pages/news_and_events/news/2014/12/news_detail_002239.jsp&mid=WC0b01ac058004d5c1, http://www.ema.europa.eu/ema/index.jsp?curl=pages/news_and_events/news/2013/06/news_detail_001835.jsp&mid=WC0b01ac058004d5c1)—of which only one uses stem cells (which is a further complication in the regulatory dossier). The cell therapy market is a highly volatile one, with volatility originating from the use of disruptive technologies, the current economic situation and the small size of the market increasing the impact of individual company fluctuations [4]. In such a market, companies as well as academic research institutes are in need of tools to advance their understanding and, at the same time, reduce their R&D cost, increase product quality and productivity, and reduce the time to market to enable the development of a customized business model for regenerative medicine.

Over recent years, concepts such as quality by design (QbD), which have long been embraced by the traditional engineering communities, are transferred to the pharmaceutical/medical field in general and the TE field in particular. As an example of this evolution, the Food and Drug Administration (FDA) of the USA now demands QbD for pharmaceutics, replacing the old adage of quality by analytics [5]. QbD involves a number of tools to control the variation of a process pre-emptively. This includes tools to measure and understand the variation in historical data as well as tools, such as *in silico* modelling, to predict, analyse and eliminate sources of variation. Traditional engineering domains have for many years adopted QbD, and *in silico* models are actively used as an integral part of the R&D pipeline and decision-making process, increasing innovation, productivity and robustness and reducing time to market. Translating this to the regenerative medicine field, the use of simulation tools could enable the incorporation of knowledge on mechanisms of action (i.e. underlying biological mechanisms) into the ATMP development pipeline, thus permitting the field to move away from the trial-and-error approach (blind screening) and increase its success rate for clinical transfer (see [6] for an example in the cancer field). Additionally, *in silico* tools should be officially recognized by regulatory bodies as an intrinsic part of the biomedical R&D pipeline. The FDA has already approved the use of *in silico* models to replace certain animal experiments in preclinical tests in the diabetes field [7] and is actively engaged in the setting up of guidelines for inclusion of modelling results in preclinical dossiers [8]. The main result of all these advantages is a cost-effective, robust and efficient approach to the development of TE products, making them commercially viable.

**Figure 1.**
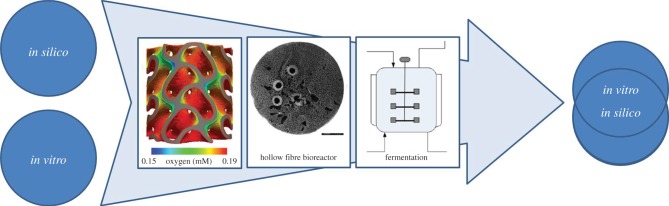
Schematic overview of the different models discussed in this paper ordered according to the level of integration of the *in silico* tools in the overall toolbox of the bioprocess field they relate to. Right: part of the fermentation set-up for insulin production [11]; middle: cross-sectional view through a hollow fibre bioreactor used for whole blood production [12]; left: oxygen levels inside neotissue grown in a three-dimensional porous scaffold in a perfusion bioreactor set-up for ATMP production (Y Guyot *et al.* 2016, unpublished work, continued from [13]).

In this paper, we discuss the use of *in silico* models in the design and production of cell-based combination products for TE. A number of works in the literature provide a comprehensive overview of the different type of models used in the context of TE [9,10]. In these works, examples are provided of the use of *in silico* models for the design of cellular differentiation protocols, biomaterials, bioreactors and overall patient treatment strategies. Rather than focusing on the TE product, the current review focuses on the process (the mantra ‘the product is the process’ is becoming more and more embedded in the TE community). The following sections discuss three (clusters of) examples related to this subject (figure 1). The first example comes from the pharmaceutical engineering field where QbD principles and their implementation through the use of *in silico* models are both a regulatory and economic necessity. The second example is related to the production of red blood cells (RBCs). The described *in silico* model is mainly used to investigate the manufacturing process of the cell-therapeutic product, and pays special attention to the economic viability of the process. Finally, we describe the set-up of a model capturing essential events in the development of a tissue-engineered combination product in the context of bone TE. For each of the examples, a short introduction to some economic aspects is given, followed by a description of the *in silico* tool or tools that have been developed to allow the implementation of QbD principles and optimal design.

## 2. Modelling pharmaceutical development and manufacturing

### 2.1. Economic considerations

The cost of therapeutic drugs is the subject of ever-increasing scrutiny by governments and the public at large [14]. In addition, the increasing number of drugs that turn out to be less effective than anticipated, or even that have dangerous side effects, has raised questions regarding risk management and manufacturing quality processes employed in the pharmaceutical industry. This has created a drive for better tools in pharmaceutical engineering with *in silico* modelling being one of them. There are several ways in which *in silico* modelling can help to improve the cost–benefit ratio of the drug discovery-to-delivery process, namely product/process R&D and manufacturing [14].

The time and cost involved in drug development is enormous: US$0.8 to 2 billion, with a yearly 8% increase of this amount, and a time to market of 10 years, which is half the patent life [15]. The three crucial stages in the development of novel drugs are (i) discovery, (ii) product/process development, and (iii) clinical trial. With the first and the last issue addressed in the Avicenna Roadmap [16], this paper will discuss a few examples of the second: product/process development. Development typically takes about 30–35% of the R&D cost and roughly 4–5 years of the duration of the entire product life from inception to launch [17]. Modelling can enhance the quantitative understanding of unit operations and materials, their performance and the integrated process perspective.

Once the process is developed, manufacturing has to take place. Again, this is a less well known but nevertheless important aspect of the economics of the pharmaceutical industry. The cost of goods sold, for instance, amounts to 27–30% for brand-name pharmaceuticals. Improving the process might therefore have a substantial effect on the cost reduction [14].

### 2.2. Process modelling

In a review paper, Gernaey & Gani [11] discuss a number of model-based systems approaches to design and analyse pharmaceutical products and/or processes. They cover constitutive models (relating intensive process variables to extensive system variations) and process (dynamic) models. Questions that are often addressed pertain to the crystallinity of powders (drugs), biosynthetic production of substances such as insulin and fermentation processes for recombinant protein production (to name but a few examples from the vast body of literature on this subject). A typical feature in these models is the combination of data-based and mechanistic modelling.

In another publication by the same group [18], the need for ontology for knowledge representation and management was addressed. The breadth of process monitoring and analysis tools for a wide range of unit operations has rendered their selection a difficult, time-consuming and challenging task. Therefore, an efficient and systematic knowledge base coupled with an inference system is necessary to support the optimal selection of process monitoring and analysis tools, satisfying the process and user constraints [18].

## 3. Modelling cell manufacturing for red blood cell production

### 3.1. Economic considerations

Every year around 92 million units of donor whole blood (a unit typically contains around 2 × 10^12^ cells) are collected globally [19], yet blood inventory shortages still occur. These shortages are particularly cumbersome in developing countries where the donor collections are largely insufficient, but even in developed countries such as the USA 7% of the hospitals report shortages causing them to postpone elective surgeries [20]. This pressure on the supply brings about unwanted risks such as the pushing into circulation of units of donated blood that have not been thoroughly screened for viruses and other transmittable diseases [19]. A potential solution to this problem is *ex vivo* blood production, which would possibly allow shortages and scale-up donations of rare blood types to be tackled. However, making this approach economically viable is a substantial challenge. An average hospital in the USA pays only US$225.42 for a typical unit of blood and US$1150 to US$3025 for a unit of rare blood [21,22]. These numbers are in stark contrast to the costs of the *ex vivo* production lines that are currently investigated. Take, for example, the process described by Giarratana *et al*. [23], the costs for the culture medium alone (including basal medium, erythropoietin (EPO), interleukin 3 and other reagents) amount to US$8330 per unit of RBCs (at 5 × 10^7^ cells ml^−1^). Even though many of the reagents can be replaced by cheaper small molecule mimetics, only the basal medium alone currently costs more than the value of a unit of RBCs mentioned above.

Recent work in bioreactor design by the group of Mantalaris [24,25] for *ex vivo* growth of whole (healthy) blood has demonstrated that a proper design, recapitulating the architectural and functional properties of blood formation, is able to reduce the need for growth factors by an order of magnitude. In contrast to two-dimensional static culture of RBCs, which is hard to scale up due to the huge amounts of medium and surface required, hollow fibre bioreactors allow the cells to grow at higher densities, reducing the required culture space and medium. Hollow fibre bioreactors continuously deliver nutrients and clear waste products through capillaries with semi-permeable membranes from the bulk (extra-capillary space) that contains the cells embedded in a scaffold that can mimic a specific three-dimensional micro-environment. The dual hollow fibre design proposed by Panoskaltsis *et al.* [25] furthermore allows recycling of the expensive growth factors in a separate circuit of capillaries. Despite the clear potential of this bioreactor, a rigorous analysis is needed to identify whether or not this set-up could be commercially viable.

### 3.2. Modelling red cell production in a parallelized hollow fibre model

Misener *et al.* [12] have developed a computational model of the above-described parallelized hollow fibre bioreactor. This model consists of a description of the biological process as well as a description of the mass transfer of a certain species in the bioreactor. For the simulation of the biological processes occurring in the bioreactor, all related to haematopoiesis, the authors built on work by Colijn and Mackey [26] and Ma *et al.* [27]. This adapted model contains a description of the kinetics of the most relevant cell types, these being haematopoietic stem cells, erythrocytes, lymphocytes and an aggregate of granulocytes and monocytes. Additionally, the model incorporates a description of the concentration of relevant metabolites, glucose, lactate and oxygen, and growth factors, stem cell factor and EPO. Mass transfer was modelled using the well-established Krogh cylinder approximation, which is suitable for hollow fibre bioreactor modelling [28]. Parameters were derived from relevant experimental studies reported in the literature. This model was subsequently used in a deterministic, global, superstructure optimization for designing and operating the bioreactor. The optimization problem was formulated as a mixed-integer nonlinear program (MINLP) and solved to deterministic global optimality using ANTIGONE [29]. The objective of the model was to design optimal bioreactor settings, allowing the *ex vivo* production of blood to become (fiscally) competitive with respect to the transfusion market for rare blood. The design and operating choices that were considered by Misener *et al.* [12] included: (i) size/aspect ratio of the cylindrical bioreactor; (ii) number of hollow fibres for delivering reactants and extracting products/by-products; (iii) flow rate of nutritious medium through the bioreactor; (iv) medium composition; and (v) oxygen concentration. The calculated global minimum for the bioreactor superstructure amounted to US$277 per unit produced, excluding the price of nutrients, bioreactor materials and fabrication, operator time, and product transportation and storage. This makes it competitive with a typical unit of rare blood. This model clearly outlines the potential for computational modelling to design not only individual bioreactors but also (and more generally) the bioprocesses.

## 4. Modelling combination products for bone tissue engineering

### 4.1. Economic considerations

Long-bone defects that result from trauma or bone-related diseases are quite common and, in general, the remarkable capacity of bone to repair itself is sufficient for the defect to heal with standard medical care. However, about 5–10% of the 14 million fractures occurring annually in Europe and the USA are associated with impaired healing, including delayed union or non-union. These fractures are responsible for a huge socio-economic burden due to the direct costs of medical care as well as the indirect costs of rehabilitation and lost productivity [30]. The gold standard treatment, autografts, suffers from limited availably of transplantable bone tissue, the considerable risk of donor-site morbidity and varying success rates [31–34], paving the way for regenerative medicine. Health economics studies indicate that regenerative therapies are beneficial for society [35–37], justifying reimbursement. The economic activity in the field of regenerative medicine and TE has grown remarkably in recent decades. Approximately 50 firms or business units offered commercial TE products with total sales above US$1.3 billion in 2007 worldwide [38,39]. The majority of the commercial sales of TE products, US$1.1 billion, was for bone and joint applications and was realized in the USA. Overall sales in bone and joint applications remained stable into 2009. From 2009 onwards, a steady increase was projected to reach US$20 billion by 2018 [3].

### 4.2. Modelling three-dimensional neotissue formation in a perfusion bioreactor set-up

According to the classical TE paradigm, TE products are composed of a combination of cells and carriers, cultured in a bioreactor environment with specific stimulation coming from the dynamic culture environment and/or the composition of the culture medium. Here, we describe the example of a model that was developed to capture the essential elements of such a classical TE product. The example pertains to the domain of bone TE and considers the culture of human periosteum-derived cells (hPDCs) [40] on regular titanium scaffolds (produced through additive manufacturing) [41] in a perfusion bioreactor set-up under various dynamic culture regimes [42]. Experimental observation of these experiments seemed to confirm the curvature-based growth principle that had been observed elsewhere in the literature [43]. Guyot *et al.* [44] explored the concept of the level-set method for simulating this curvature-dependent neotissue (cells and their extracellular matrix) growth by tracking the evolution of the interface between the neotissue and the void space on the three-dimensional scaffold. An *in silico* study was conducted, recapitulating a previous experiment [41] in which different scaffold geometries (hexagonal, triangular and square) and scaffold pore sizes (500 and 1000 µm) were seeded with hPDCs and cultured under static conditions for 14 days. A qualitative and quantitative comparison was carried out between the experimental data and the numerical results based on the projected tissue area, and a good correlation between both was demonstrated, showing the relevance of the model. Subsequently, the model was extended to simulate the dynamic culture conditions with the help of the Brinkman equations governing the flow, to investigate the local shear stresses that cells are exposed to during the culture process [13]. This addition of equations governing the fluid velocity profile both in the culture medium as well as in the growing neotissue (considered as a porous medium) constituted a novelty in modelling neotissue growth under dynamic conditions. Indeed, most of the studies reported in the literature focus on calculating the shear stress on empty scaffolds [45,46] or consider the neotissue as an impermeable volume where no flow is allowed [47–49]. The aforementioned approaches might be sufficient for capturing the initial stages of neotissue formation. But they are inadequate for capturing the later stages as the growing neotissue is a porous tissue that allows flow through its own micro-pores, thereby changing its surrounding flow environment as well as the mechanical (shear) stimulation on the cells inside the neotissue. Results presented in Guyot *et al.* [13] show the ability of the developed model to tackle this issue by calculating the level of shear stress not only on the interface between the neotissue and the culture medium (void space), but also within the biological construct itself, where an approximation based on the computed interstitial fluid velocity was made. The computed inner shear stress was around 10–50 times higher than the surface shear stress, which demonstrates the influence of the progressing neotissue–void interface and the porous nature of the neotissue on the results obtained with the fluid equations. Next, the model was extended to take into account the influence of the computed shear stress on the definition of the neotissue growth velocity [50]. Since the mechanical stimuli that cells are exposed to have been shown to dramatically affect the cells' behaviour and proliferation, an expression depicting the shear stress stimulatory effect for moderate shear stress values and its detrimental effect on high values were added into the neotissue growth velocity definition (based on work by Nava *et al.* [48] and Chapman *et al*. [51]). The updated model was able to clearly demonstrate the effect of shear stress on neotissue growth by simulating growth under two different flow rates and comparing the results with in-house experimental data. Even though there was an imperfect match between the exact neotissue growth kinetics between experiments and simulations, the model was able to largely capture the difference in growth between the different flow rates. In the next version of the model (Y Guyot *et al.* 2016, unpublished data), partial differential equations representing metabolic activity (including oxygen, glucose, lactate and pH) were incorporated in order to capture their effect (whether negative or positive) on neotissue growth velocity. After calibration of the model by comparing the computed neotissue volume fraction with the experimentally obtained values for two in-house-produced scaffold designs, the model has been used to optimize the culture process (scaffold design and culture conditions) to maximize the neotissue growth speed. Validation experiments are currently ongoing. In its final version, this model will be a tool for intelligent scaffold geometry design where several designs can be tested *in silico* in order to find the best candidate for three-dimensional cell growth guidance, or a tool for controlling the flow-induced shear stress on cells by varying the flow rate *in silico* and selecting the optimum fluid velocity; it will also contribute to a better understanding of the local effect of metabolite concentrations on neotissue growth. Knowing with precision the local concentration of nutrients and waste products within the scaffold, and particularly in the neotissue, provides the bioprocess operator with a much higher degree of insight and control which will translate into an enhanced quality of the final construct while permitting rigorous optimization based on biological and economic arguments. The use of such tools is required to bring the field of TE closer to robust and reliable clinical translation.

## 5. Conclusion

This review discussed examples of *in silico* models in different domains of the field of regenerative medicine, ranging from pharmaceutical engineering, to cell production to ATMP-combination product development. In each of these domains, *in silico* modelling is in a different stage of embeddedness in the product and process R&D pipeline. *In silico* models were shown to be of substantial added value to address both regulatory and financial issues. Both the regulatory and the financial drivers will ultimately be instrumental in the role that *in silico* tools will have to play in the development of a viable business model for regenerative medicine applications.
